# *Helicobacter pylori* outer membrane vesicles mediate central tolerance in C57BL/6J mice offspring T cells via maternal-fetal transmission

**DOI:** 10.3389/fimmu.2025.1522842

**Published:** 2025-04-15

**Authors:** Yusen Wei, Lu Zhou, Xiaofei Zhao, Huiqing Qiu, Dailun Hu, Zhongli Shi

**Affiliations:** ^1^ Graduate School, Hebei Medical University, Shijiazhuang, China; ^2^ Department of Oncology, Hebei General Hospital, Shijiazhuang, China; ^3^ Department of Pharmacy, Hebei General Hospital, Shijiazhuang, China; ^4^ Department of Neurology, The First Hospital of Hebei Medical University, Shijiazhuang, China; ^5^ Department of Pathogenic Biology, Hebei Medical University, Shijiazhuang, China; ^6^ Hebei Key Laboratory of Brain Science and Psychiatric-Psychologic Disease, The First Hospital of Hebei Medical University, Shijiazhuang, China

**Keywords:** *Helicobacter pylori* (*H.pylori*), outer membrane vesicles (OMVs), pregnancy, T cells, central tolerance

## Abstract

Outer membrane vesicles (OMVs) released by *Helicobacter pylori* (*H.pylori*) can enter the blood circulation of the host and cause extra-gastric lesions such as atherosclerosis and hyperemesis gravidarum. This study aimed to investigate the effect of OMVs released by *H.pylori* on the development of thymic T cells in offspring mice and its underlying mechanisms. Through experimental observations, we found that *H.pylori* OMVs were able to cross the placental barrier, leading to a decrease in the number of CD3^+^ and CD4^+^ T cells in the peripheral blood of the offspring mice and a decrease in the response of T cells to *H.pylori* stimulation. After stimulation with OMVs in T cell positive selection experiments, the expression levels of CHMP5, IKK-β, and NF-κB are up-regulated, and the release of cytokines IL-7, IL-2, IL-4, and IFN-γ is simultaneously increased, whereas in T cell negative selection experiments, the expression of JNK is up-regulated, and the expression of CHMP5 and Bcl-2 is down-regulated in E15-16 fetal thymus organ culture. These results indicate that transmission of *H pylori* OMVs from mother to fetus might be related to the development of central tolerance in offspring T cells. The underlying mechanism may involve an interaction between the OMVs-stimulated pathway and the TCR pathway, although further research is needed to confirm this hypothesis. The study highlights the importance of preventing *H.pylori* infection during pregnancy and suggests that the effect of centrally tolerated antigens needs to be considered in vaccine design to maximize prevention.

## 1 Introduction

*Helicobacter pylori* (*H pylori*) is a gram-negative bacterium that can colonize the stomach for a long time (1–3). About half of the world’s population is infected with *H pylori* (4, 5), and nearly half of pregnant women are also infected (6, 7). Severe *H pylori* infection in pregnant women has been reported to lead to abortion and developmental malformations (8, 9). However, the effect of *H pylori* infection during pregnancy on fetal development remains poorly understood. This gap in knowledge may be due to the perception that bacteria cannot pass through the placental barrier.

The outer membrane vesicles (OMVs) released by *H pylori* play a crucial role in its pathogenesis (10–12). OMVs are key mediators of bacterial-host interactions, capable of delivering bacterial proteins and other molecules into host cells, thereby affecting the host’s immune response and cellular function (13). Recent studies have shown that OMVs can enter the host’s circulatory system and may cross the placental barrier (14). This raises concerns about the potential effects of *H pylori* infection during pregnancy on the fetus.

Although the placental barrier provides a degree of protection to the fetus from harmful substances, it can be compromised by certain pathogens (15, 16). These pathogens can cross the placental barrier and infect the fetus, leading to various pregnancy complications. Therefore, understanding the integrity of the placental barrier is essential to protect the fetus from infection. However, relatively little research has been conducted on whether *H pylori* OMVs can cross the placental barrier and affect fetal health. This area of research is important for understanding the maternal-fetal transmission of *H pylori* infections and their potential impact on offspring health.

Our central hypothesis is that *H pylori* OMVs can cross the placental barrier and affect fetal immune system development, particularly the development of thymic T cells. The key research questions we aim to address are: (1) How do *H pylori* OMVs cross the placental barrier? (2) What are the effects of *H pylori* OMVs on the development of thymic T cells in offspring? (3) What are the underlying mechanisms by which OMVs influence T cell development?

To address these questions, we examined the effects of *H pylori* OMVs on the development of thymic T cells in offspring mice. Specifically, we assessed the impact of OMVs on the number of CD3^+^ and CD4^+^CD8^-^ T cells in the peripheral blood of offspring mice, analyzed T cell responses to *H pylori* stimulation, described changes in molecular and cytokine expression in positive T cell selection assays, and discussed changes in molecular expression in negative T cell selection assays. We hope to provide new insights into the effects of *H pylori* infection on fetal immune system development during pregnancy.

**Figure 1:**
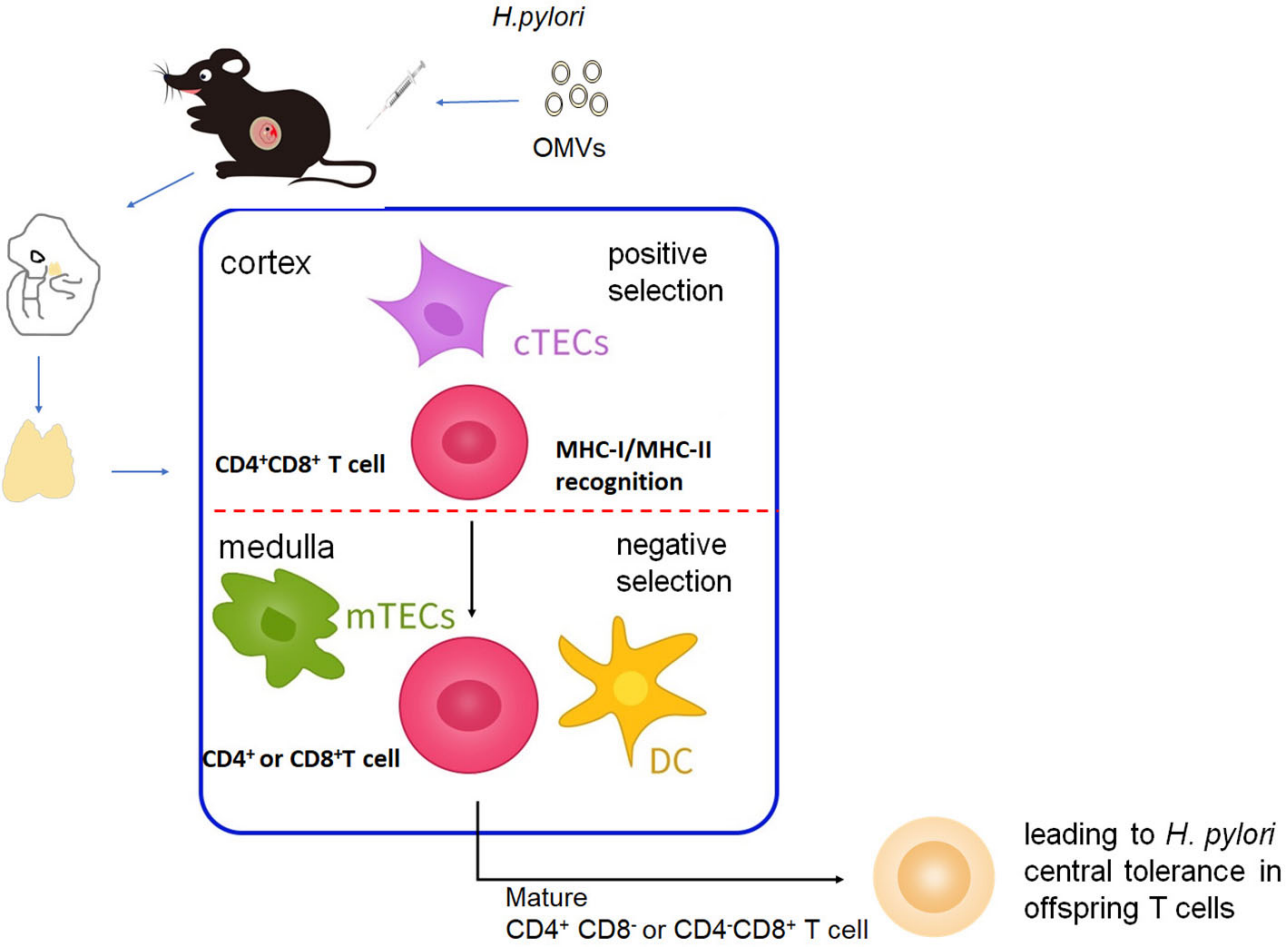
Schematic representation of *H pylori* OMVs mediating central tolerance of T cells from C57BL/6J mice offspring via maternal-fetal transmission.

The significance of this study is to reveal the impact of maternal-fetal transmission of *H pylori* OMVs on the central tolerance of offspring T cells (**Figure 1**), elucidate the mechanism of interaction between the OMVs-stimulated pathway and the TCR pathway. We highlight the importance of screening for *H.pylori* infection, especially during pregnancy, and increasing awareness among women of child-bearing age. While it may be challenging to completely prevent *H.pylori* infection, these results emphasize the need for proactive measures to identify and manage the infection during this critical period, as well as considerations regarding centrally tolerated antigens in vaccine design, and potential approaches to maximize the effectiveness of prevention.

## 2 Methods and experimental models

### 2.1 *H.pylori* culture and OMVs extraction and purification

According to previous studies, we made appropriate modifications (17). The *H.pylori* SS1 (purchased from BioVector NTCC Inc., cat.no. Hp SS1) used was resuscitated in Columbia solid medium containing 10% defibrinated sheep blood and transferred to brain heart infusion (BHI) medium (containing 10mg/L vancomycin, 2.5mg/L amphotericin B, 5mg/L trimethoprim, and 2.5IU/L polymyxin B, all antibiotics were purchased from Sigma-Aldrich, Germany). After microaerobic culture (85% N_2_, 10% CO_2_, 5% O_2_) at 37°C for 3-5 days under shaking at 120rpm/min, the supernatant was collected by centrifugation at 2000g for 10 min at 4°C, filtered with 0.45 and 0.22μm membranes, respectively, and then centrifuged with 100kDa (Millipore, USA) ultrafiltration tube at 3000g for 10min at 4°C. The pure OMVs were then extracted through an exclusion column kit (Invitrogen exosome spin column MW3000, USA) according to the manufacturer’s instructions (18). Protein concentrations of OMV preparations were quantified using the BCA Protein Assay Kit (Pierce Thermo Scientific, Rockford, IL, USA).

### 2.2 Identification of OMVs

Using the NanoSight^®^ NS300 Nanoparticle Tracking Analysis (NTA) device, extracted OMV samples were diluted at a ratio of 1:100 and measured to obtain information on the size, and distribution of the particles in the samples (18).In addition, the samples were dropped onto FCF300-Cu grids (Sigma-Aldrich, Germany), fixed with 1% glutaraldehyde for 5min, dried, observed in a transmission electron microscope, and photographed (Hitachi HT7800, Japan) (19). The samples were then mixed with loading buffer and subjected to sodium dodecyl sulfate polyacrylamide gel electrophoresis (SDS-PAGE) at 95°C for 10min. After being rapidly stained with Coomassie Brilliant blue (R-250), the samples were identified by gel digestion and mass spectrometry.

### 2.3 Protein extraction and analysis of *H.pylori* and OMVs

The bacteria were collected by centrifugation (5000r/min, 4°C), washed 3 times with precooled PBS, lysed with protein lysis buffer containing protease inhibitors and nucleases, mixed, and incubated on ice for 30min. The suspension was treated by sonication (3s sonication, 9s interval, cooling by ice bath) until the cells had broken. The precipitate was then discarded by centrifugation (12000r/min, 4°C), and the supernatant was transferred to Eppendorf tubes. Protein was quantified by BCA method.

The extracted *H.pylori* whole-cell protein and OMVs samples were mixed with loading buffer, respectively, subjected to SDS-PAGE at 95°C for 10min in a water bath, and then transferred to a polyvinylidene difluoride (PVDF) membrane. The membranes were blocked with 5% skim milk powder in TBST for 1h and then incubated overnight at 4°C with the primary antibody (a polyclonal antibody obtained from cows immunized with inactivated whole SS1 bacteria by our research group). After a wash step, the membranes were incubated with the secondary antibody [goat anti-bovine IgG (H+L) HRP, Invitrogen, 1:500 dilution] for 1h at room temperature. Finally, an imaging system (Amersham Imager 600, GE Healthcare, Britain) was used to observe the membranes and to take pictures. The stained bands were subsequently dug out and identified by Coomassie Brilliant blue tape in-gel digestion and mass spectrometry.

### 2.4 Cell culture of BMDCs and *H.pylori* antigen extracted by BMDCs

Bone marrow (BM) was washed from the femur and tibia of mice. After purification, the cell concentration was adjusted to 1×10^6^ cells/mL. The cells were plated onto 100mm bacterial culture dishes with RPMI 1640 complete medium containing 10% FBS and GM-CSF (20ng/mL, Abcam, ab9742). Another 10mL fresh medium containing 20ng/mL GM-CSF was added to the culture dish. On day 6 and day 8, the old medium was collected and centrifuged, and the cells were resuspended in fresh medium containing 20 ng/mL GM-CSF, before being returned to their original dish. Cells were harvested at day 10 and identified by flow cytometry (20, 21).

Mature BMDCs (1×10^7^ cells/mL) were incubated with PBS, *H.pylori*, and *H.pylori* OMVs for 5 days at 1000 r/min, and the cells were collected by centrifugation. Subsequently, 4mL weak acid extract [0.131mol/L citric acid, 0.066mol/L disodium hydrogen phosphate (Sigma-Aldrich, USA)] was added, and the pH was adjusted to 3.3. The supernatant was shaken gently at room temperature for 5min, aspirated by centrifugation, and then intercepted by centrifugation in a 3kDa (Millipore, USA) ultrafiltration tube at 3000g for 10min at 4°C, and then analyzed by mass spectrometry (22).

### 2.5 Mass spectrometry analysis

Sample preparation for mass spectrometry began with extraction of the target protein bands from the gel and washing with MilliQ water, followed by treatment with a decolorizing solution of ammonium bicarbonate and acetonitrile for 20 min at 37°C to remove the pigmentation until the color disappeared. The samples were then dehydrated, vacuum dried, and treated with DTT reduction and IAA alkylation. Afterwards, the micelles were eluted using a solution of ammonium bicarbonate and acetonitrile and fixed by vacuum drying. After addition of trypsin buffer solution, the samples were incubated at 4°C for 30 min, followed by overnight digestion at 37°C. The digestion reaction was terminated with 0.1% trichloroacetic acid and the digest was collected by centrifugation. Trypsin-digested peptides were analyzed using a Q Exactive HF-X mass spectrometer and an EASY-nLC 1200-Q Exactive HF-X system, and NCBI databases were searched via the MASCOT search engine (Matrixscience.com) for protein identification.

### 2.6 Mice experiments

All animal experimental protocols were implemented after approval by the Animal Ethics Committee of the First Hospital of Hebei Medical University, and all animals reached the SPF level that was assured as being non-infected by *H.pylori*. C57BL/6J mice were housed in a pathogen-free room (12 h day/night, temperature 21 ± 2°C, humidity 55 ± 10%) and fed AD libitum. The *H.pylori* infection model was established as follows (23): freshly cultured *H.pylori* was collected, and the concentration of bacterial solution was adjusted to 1×10^9^ CFU/mL. Three to four weeks old female mice were selected. In order to achieve *H.pylori* colonization, 0.1 mL of 50% alcohol was given to each mouse by gavage one day before inoculation. (18). *H.pylori* was given by gavage, 0.2mL each time, once every other day, 3 times. All animals were fasted and water-deprived for 12h before feeding and for an additional 4h after feeding. The mice were caged (vaginal plug dates were designed as E1), and the peripheral blood of the offspring mice was collected to analyze the changes of peripheral blood T cell subsets in the offspring mice when they were 6-8 weeks old.

An OMVs model for tail vein injection was established: OMVs were resuspended in sterile PBS, incubated with 5μM DiI (1,1’-dioctadecyl-3,3,3’,3’-tetramethylindocarbocyanine perchlorate, Invitrogen, USA) for 30min, then washed three times to remove impurities such as free DiI and lipoproteins, and resuspended in PBS (24, 25). Pregnant E18-19 mice were injected with DiI-labeled *H.pylori* OMVs (0.2mL, 20μg/mL) via the tail vein, and Cy5-IgG was injected as a positive control. Offspring were born, and the fluorescence signal of the offspring was detected within 24h by using the FX Pro system (Bruker, USA). Only pregnant mice with 3-6 fetuses were included in this study.

### 2.7 Positive-selection experiments and negative-selection experiments

The positive and negative selection experiments were appropriately adapted according to the guidance of the literature (26, 27). The experimental steps were as follows: first, pregnant mice (embryonic day E15-E16) were executed and their abdomens were wiped with 70% ethanol. Next, the abdominal wall was incised to expose the uterus, which was carefully incised with scissors and forceps to remove the embryos and to strip the placenta and fetal membranes. The removed embryos were then placed in petri dishes containing RF10-H medium.

For positive and negative selection experiments, thymic lobes were isolated from embryos and transferred to Nucleopore polycarbonate filters (Costar). Thymic lobes were cultured at 37°C with 5% CO_2_ using RP10 medium (RPMI1640 medium supplemented with 10% v/v heat-inactivated FCS, 2 mM glutamine, 10 mM HEPES, 0.5 mg/mL folate, 0.2 mg/mL glucose, 100 U/mL penicillin, and 100 μg/mL streptomycin).

For positive selection experiments, fetal thymus organ culture (FTOC) was established using thymic lobes from E15-E16 embryos. *H pylori*-OMVs at a concentration of 0.4 μg/mL were added at the start of the culture process, and the culture was maintained for 24 hours. For the negative selection experiments, FTOC was also established using thymic lobes from E15-E16 embryos. After 3 days of incubation, the thymic lobes were transferred to a medium containing 0.4 μg/mL of *H pylori* OMVs, and incubation was continued for an additional 24 hours.

Finally, thymocytes that underwent positive and negative selection were collected separately. After gently hand-damaging the thymus blades in 1.5 mL Eppendorf tubes, they were analyzed by flow cytometry. These steps were designed to simulate and study the development and differentiation process of thymocytes under different selection pressures.

### 2.8 Flow cytometry

E15-E16 pregnant mice (9-week-old, 30-33g) were used. After the fetal mice thymic lobes had been dissected, one half of each lobe was cultured for 4 days (control group), and three groups of duplicate wells were set up. The other half of each of the thymic lobes was cultured for 3 days and then stimulated with *H.pylori* OMVs for 1 day (experimental group), and 3 groups of duplicate wells were set up. On the fourth day, fetal thymus CD4 and CD8 were analyzed by flow cytometry.

The peripheral blood of 6- to 8-week-old offspring mice was collected, with 4 mice per group. To each 100 μL of whole blood, 30 μL of heparin sodium solution was added to prepare anticoagulated blood. Antibodies CD3 (PE, no.100206), CD4 (FITC, no.100510), and CD8 (PerCP/Cyanine5.5, no.100734) were added to the anticoagulated blood. Antibodies were purchased from BioLegend. The cells were incubated in the dark for 30min. Subsequently, 2mL red blood cell lysate was added to the cells and mixed gently. The reaction was carried out at room temperature in the dark for 10min. The samples were centrifuged at 1500rpm for 5min, and the supernatant from each was discarded. After being mixed with 2mL PBS, the cells were centrifuged at 1500rpm for 5min, the supernatant was discarded, and the cell precipitate was resuspended in 500uL of PBS and examined by flow cytometry.

All experiments included the addition of a mice Fc receptor blocker to perform blocking operations to avoid non-specific staining. The results were analyzed using Attune NxT software (Invitrogen, USA), and the gate strategies are shown in **Supplementary Figure**.

### 2.9 Mixed lymphocyte reaction

To detect cell proliferation (21), we first co-incubated mature bone marrow-derived dendritic cells (BMDCs, 1×10^5^/well/100uL) with *H pylori* for 5 days. At the same time, peripheral blood samples were collected from the offspring of mice injected with *H pylori* OMVs via tail vein in late gestation (E18-E19). Monocytes were isolated from these samples using isodensity gradient centrifugation and B lymphocytes were cleared by CD19 magnetic beads (Meteni, Germany). Subsequently, purified BMDCs were co-cultured with T cells at a ratio of 1:10 for 5 days. At the end of the culture, cell culture supernatants were collected for subsequent cytokine assays.

### 2.10 Cellular immunofluorescence

The cell source is the same as that of the mixed lymphocyte reaction experiment. Cells were fixed with 4% paraformaldehyde (PFA) for 15min at room temperature, permeabilized with 0.3% Triton X-100, blocked with 2% goat serum for 1h, and incubated with rabbit anti-Ki-67 (Invitrogen, MA5-14520 at 1:250 dilution) overnight at 4°C. The cells were subsequently incubated with Alexa Fluor 555-conjugated secondary antibody goat anti-rabbit IgG (Invitrogen, A32732, 1:200 dilution) 1 h. After a washing step, coverslips were carefully placed over the cells and sealed using an anti-fluorescence quencher. Images were acquired by confocal microscopy (Zeiss, Oberkochen, Germany) for fluorescence imaging.

### 2.11 Western blot analysis

The gels and PVDF membranes were cut according to the size of the membrane transfer mold, and the proteins were transferred to the PVDF membranes (Millipore, USA) in a Bio-Rad transfer tank. The PVDF membranes were removed and immersed in blocking solution (3% bovine serum albumin) for 1h at room temperature with shaking. Goat anti-CHMP5 antibody (GeneTex, GTX106692, 1:500 dilution), goat anti-NF-κB antibody (Abcam, ab32536, 1:1000 dilution), and goat anti-IKK-β antibody (Abcam, ab124957, 1:1000 dilution) were added to the membranes, with β-actin being used as control. The membranes were incubated at room temperature for 1h with shaking, after which time they were removed and washed 4 times with PBS for 10min each. They were then immersed in the secondary antibody solution (goat anti-rabbit IgG Dylight 800, diluted 1:5000), incubated for 1h with slow shaking, and washed 4 times with PBS for 10min each. The target protein spots were detected by an ECL colorimetric kit. Image J software was used for analysis and processing.

### 2.12 Real-time fluorescent quantitative polymerase chain reaction

Total RNA was extracted from tissues according to the Trizol (Invitrogen 15596-026) instructions (28), and its integrity was confirmed by agarose gel electrophoresis. RNA concentration and purity were determined by a Nano Drop 1000 spectrophotometer (DeNovix, USA). cDNA was synthesized by a cDNA Reverse Transcription Kit (Thermo Fisher Scientific, Waltham, MA), followed by a Maxima SYBR Green qPCR Master Mix (Thermo Fisher Scientific, Waltham, MA) to amplify the genes of interest. The primer sequences for qRT-PCR are shown in **Table 1**.

**Table 1:** The primer sets used for the detection of murine genes by qRT-PCR analysis.

Gene assayed	Primer sequence (5'–3')
CHMP5	5'-GATGAGAGAGGGTCCTGCTAAG-3' 5'-CAGGTTGTCTCGCTGTTGCTCA-3'
JNK	5'-CGCCTTATGTGGTGACTCGCTA-3' 5'-TCCTGGAAAGAGGATTTTGTGGC-3 '
Bcl-2	5'-CCTGTGGATGACTGAGTACCTG-3 ' 5' -AGCCAGGAGAAATCAAACAGAGAGG-3 '
GAPDH	5'-CATCACTGCCACCCAGAAGACTG-3' 5'-ATGCCAGTGAGCTTCCCGTTCAG-3'

### 2.13 Enzyme-linked immunosorbent assay

Cytokines were detected with IL-7, IL-2, IL-4, and IFN-γ ELISA kits, purchased from R&D (USA), according to the manufacturer’s instructions (29). First, the capture antibody was encapsulated in a 96-well plate and incubated at 37°C overnight. Subsequently, the plates were closed for one hour, samples and standards were added and incubated for 2 hours, followed by the addition of the detection antibody and substrate and incubation for another 2 hours. Afterwards, the affinity hormone-HRP coupling was added and incubated for 30 minutes. Finally, 2,2’-azathiazoline [3-ethylbenzothiazoline-6-sulfonic acid]-diammonium salt solution was added and the optical density was measured at 405 nm. Between each step, the substrate was washed thoroughly using wash buffer to remove unbound substrate. The experimental procedure for each group was repeated three times.

### 2.14 Statistical analysis

Data are presented as mean±standard deviation (SD). GraphPad Prism 9.0 was used for statistical analysis. The Shapiro - Wilk test was employed to assess the data distribution. The results indicated that the data followed a normal distribution (*P* > 0.05). Student’s t test was used for paired pairs, the two-tailed t test was used for unpaired pairs, and a one-way analysis of variance (ANOVA) and Tukey’s multiple comparison test were used for multiple group comparisons. Statistical results were expressed as *P < 0.05, ** *P* < 0.01, *** *P* < 0.001.

## 3 Results

### 3.1 Extraction and identification of *H.pylori* OMVs

**Figure 2:**
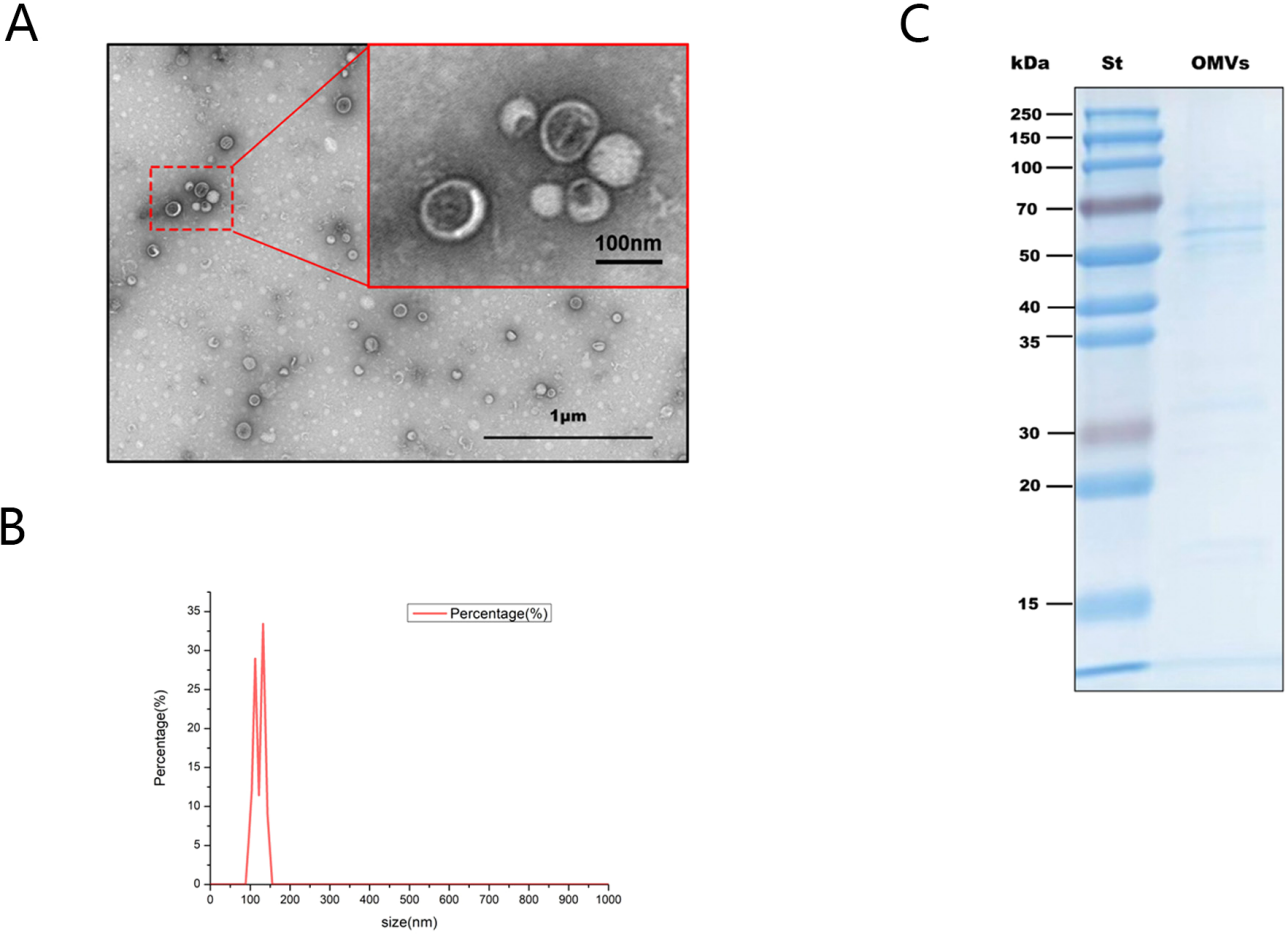
Identification of *H.pylori* OMVs. **(A)**, Typical spherical vesicle-like morphological structures with diameters between 20 and 400nm were observed by TEM. Scale bars:1 micr/on (black box) and 100 nm (red box). **(B)**, Nanoparticle tracking analysis shows the average concentration (vesicles/mL) of OMV of a specific size (nm). **(C)**, Coomassie blue staining results of *H.pylori* OMVs after SDS-PAGE electrophoresis. St indicates the molecular marker standard (kDa, left).

*H.pylori* OMVs were isolated from *H.pylori* culture supernatant and identified. Transmission electron microscopy (TEM) showed that the isolate contained spherical vesicular structures (**Figure 2A**). Nanoparticle tracking analysis (NTA) results showed that the particle size distribution of OMVs ranged from 100 to 150 nm, which was the typical particle size of OMVs produced by Gram-negative bacteria (**Figure 2B**). The extracts were subjected to SDS-PAGE, gel staining, band collection (**Figure 2C**), and in-gel enzymatic digestion followed by proteomic analysis; 343 *H.pylori* proteins were identified, including outer membrane protein (OMP), vacuolating cytotoxin A (VacA), flagellin A (FlaA), and urease (Ure) etc (**Table 2**).

**Table 2:** Proteomic analysis of *H pylori* OMVs by LC-ESI-MS/MS. This table briefly summarizes *H pylori* SS1proteins identified by mass spectrometry.

Accession	Description	Coverage [%]	Peptides	PSMs	Unique Peptides	Protein Groups	AAs	MW [kDa]	calc. pI	Peptides (by Search Engine): Mascot	Abundance: F14: Sample
A0A1U9ITG9	Outer membrane protein HopQ / Omp27 OS=Helicobacter pylori SS1 OX=102617 GN=hopQ PE=4 SV=1	43	19	30	8	1	638	69.4	9.07	19	12909955.75
A0A1U9IVK7	Vacuolating cytotoxin autotransporter OS=Helicobacter pylori SS1 OX=102617 GN=vacA PE=4 SV=1	35	30	33	26	1	1312	141.9	8.75	30	225306407.5
A0A1U9IUB7	Flagellin OS=Helicobacter pylori SS1 OX=102617 GN=flaA PE=3 SV=1	61	23	59	23	1	510	53.2	6.43	23	162104462.9375
A0A1U9IS01	Urease subunit beta OS=Helicobacter pylori SS1 OX=102617 GN=ureB PE=3 SV=1	77	36	153	36	1	569	61.6	6.01	5517	2135599392.65625

### 3.2 *H.pylori* OMVs can be transmitted across the placental barrier to the embryo

**Figure 3:**
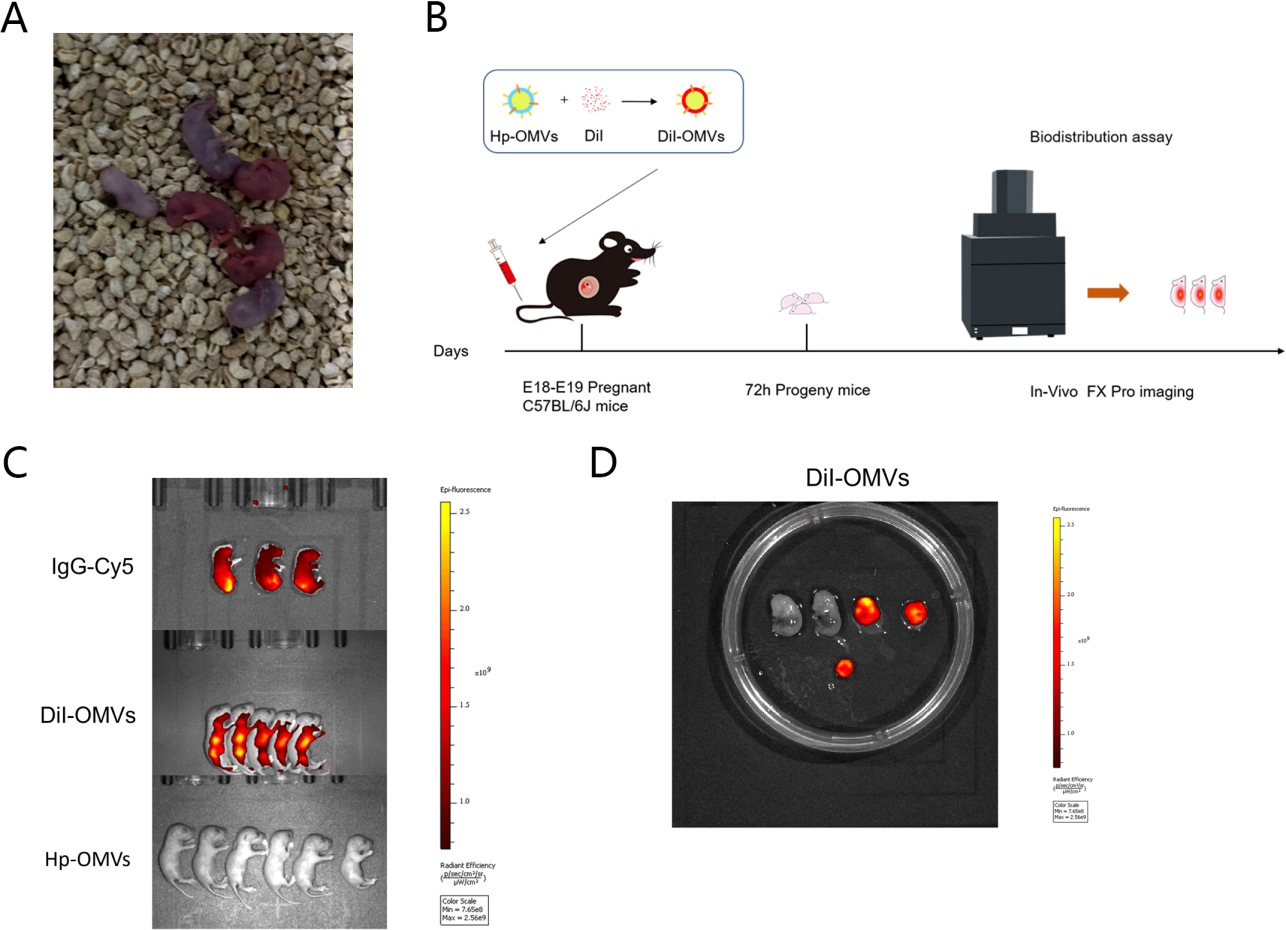
*H.pylori* OMVs is able to cross the placental barrier. **(A)**, When the concentration of *H.pylori* OMVs was greater than 40 μg/mL, vaginal bleeding and abortion were observed in E18-19 pregnant mice injected via the tail vein. **(B)**, Flow chart of DiI-OMVs administered via the tail vein to E18-E19 pregnant mice. **(C)**, Views after FX PRO (Bruker) imaging. Hyperfluorescence signal in fetal mice (upper) via tail vein injection of IgG-Cy5. DiI-OMVs were injected via the tail vein, and high fluorescence signal was observed in the offspring (lower) (right, scale bar). **(D)**, Ex vivo fluorescence imaging of mice placenta in DiI-OMVs groups (right, scale bar).

An appropriate dose of *H.pylori* OMVs was determined by a dose-effect experiment. E18-19 pregnant mice were injected with 160, 80, 40, 20, or 10μg/mL *H.pylori* OMVs (0.2mL) via the tail vein. When the concentration was higher than 40μg/mL, the pregnant mice showed premature delivery, vaginal bleeding, and abortion (**Figure 3A**); when the injection concentration was 20μg/mL, the pregnant mice had no vaginal bleeding, and the offspring mice survived for more than one year and were able to reproduce. Therefore, the 0.2mL(20μg/mL) dose was used as the experimental injection dose.

To evaluate whether *H.pylori* OMVs were able to pass through the placental barrier to the embryo. *H.pylori* OMVs were labeled with DiI and injected into E18-19 pregnant mice via the tail vein. Strong fluorescence signals were observed in the offspring mice, indicating that *H.pylori* OMVs had passed through the placental barrier. Similarly, strong autofluorescent signals were detected in isolated placentas of the DiI-OMVs group but not in offspring mice from the *H.pylori* OMVs group (**Figures 3B-D**).

### 3.3 *H.pylori* OMVs and *H.pylori* maternal bacteria have common antigens

**Figure 4:**
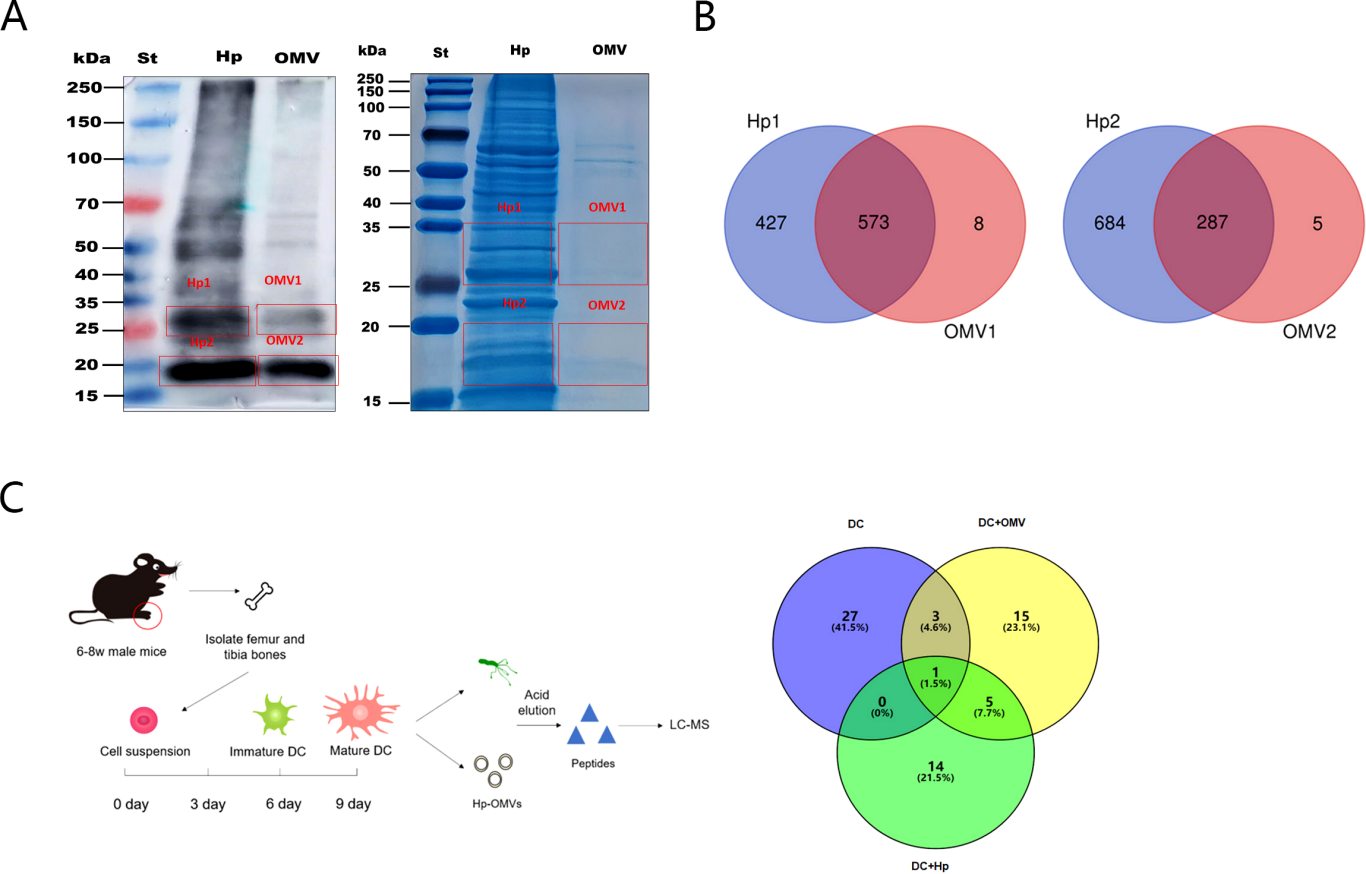
*H.pylori* OMVs carry the same antigen as *H.pylori*. **(A)**, Immunoblotting plots of *H.pylori* and of *H.pylori* OMVs show high gray values at both 25-35kDa and 15-20kDa. **(B)**, Gel stained with Coomassie brilliant blue at the corresponding positions. Venn diagram shows the amount of protein detected in whole bacteria and OMVs. **(C)**, Mature BMDCs were cultured and stimulated with *H.pylori* or with *H.pylori* OMVs. MHC peptides were eluted with weak acid and identified by mass spectrometry. Venn diagram showing antigenic peptides extracted from OMVs and *H.pylori* by BMDCs.

To analyze the antigenic components carried by the OMVs, the lysates of *H.pylori* and of *H.pylori* OMVs were subjected to SDS-PAGE and Western blot analysis. We compared the protein composition of *H.pylori* OMVs with that of *H.pylori*. The results showed that *H.pylori* and their OMVs had high gray values at 25-35kDa and 15-20kDa. The gels were cut at the corresponding positions and analyzed after in-gel digestion (**Figure 4A**), We found 1000 proteins in the 25-35kDa band of *H.pylori* whole bacteria and 581 proteins in *H.pylori* OMVs, of which 573 proteins were shared by *H.pylori* and their OMVs. We detected 971 proteins in *H.pylori* whole bacteria at the 15-20kDa band and 292 proteins in *H.pylori* OMVs, of which 287 proteins were shared by *H.pylori* and their OMVs (**Figure 4B**).

Subsequently, we co-cultured the induced mature bone-marrow-derived dendritic cells (BMDCs) with *H.pylori* or their OMVs for 5 days (**Figure 4C**). The MHC-binding antigenic peptides were then eluted under weak acid conditions, and the eluate was used for peptide identification by liquid chromatography-mass spectrometry (LC-MS/MS). The results showed that *H.pylori* and their OMVs shared six common antigenic peptides (**Table 3**).

**Table 3:** BMDCs extracted the same antigenic peptides from *H pylori*-OMVs and *H pylori*.

Accession	Description	Coverage [%]	Peptides	PSMs	Unique Peptides	Protein Groups	AAs	MW [kDa]	calc. pI	Peptides (by Search Engine): Mascot	Abundance: F7: Sample
A0A1Y3DZ56	Uncharacterized protein OS=Helicobacter pylori SS1 OX=102617 GN=X568_06790 PE=4 SV=1	9	1	2	1	1	66	7.7	9.94	1	
A0A1U9IVK7	IS607 resolvase OrfA OS=Helicobacter pylori SS1 OX=102617 GN=HPYLSS1_00302 PE=4 SV=1	3	1	1	1	1	217	25.1	9.03	1	13230317
A0A1Y3E1L4	Plasmid stabilization protein OS=Helicobacter pylori SS1 OX=102617 GN=X568_02515 PE=4 SV=1	1	1	1	1	1	543	61.9	9.16	1	6487167
A0A1U9IS95	Protein RecA OS=Helicobacter pylori SS1 OX=102617 GN=recA PE=3 SV=1	2	1	1	1	1	347	37.6	5.66	1	
A0A1U9IT63	GMP reductase OS=Helicobacter pylori SS1 OX=102617 GN=guaC PE=3 SV=1	2	1	1	1	1	325	35.9	8.53	1	
A0A1U9ITD1	Protein translocase subunit SecA OS=Helicobacter pylori SS1 OX=102617 GN=secA PE=3 SV=1	1	1	1	1	1	865	99	5.91	1	200291.171875

### 3.4 *H.pylori* OMVs can induce central tolerance of progeny T cells

**Figure 5:**
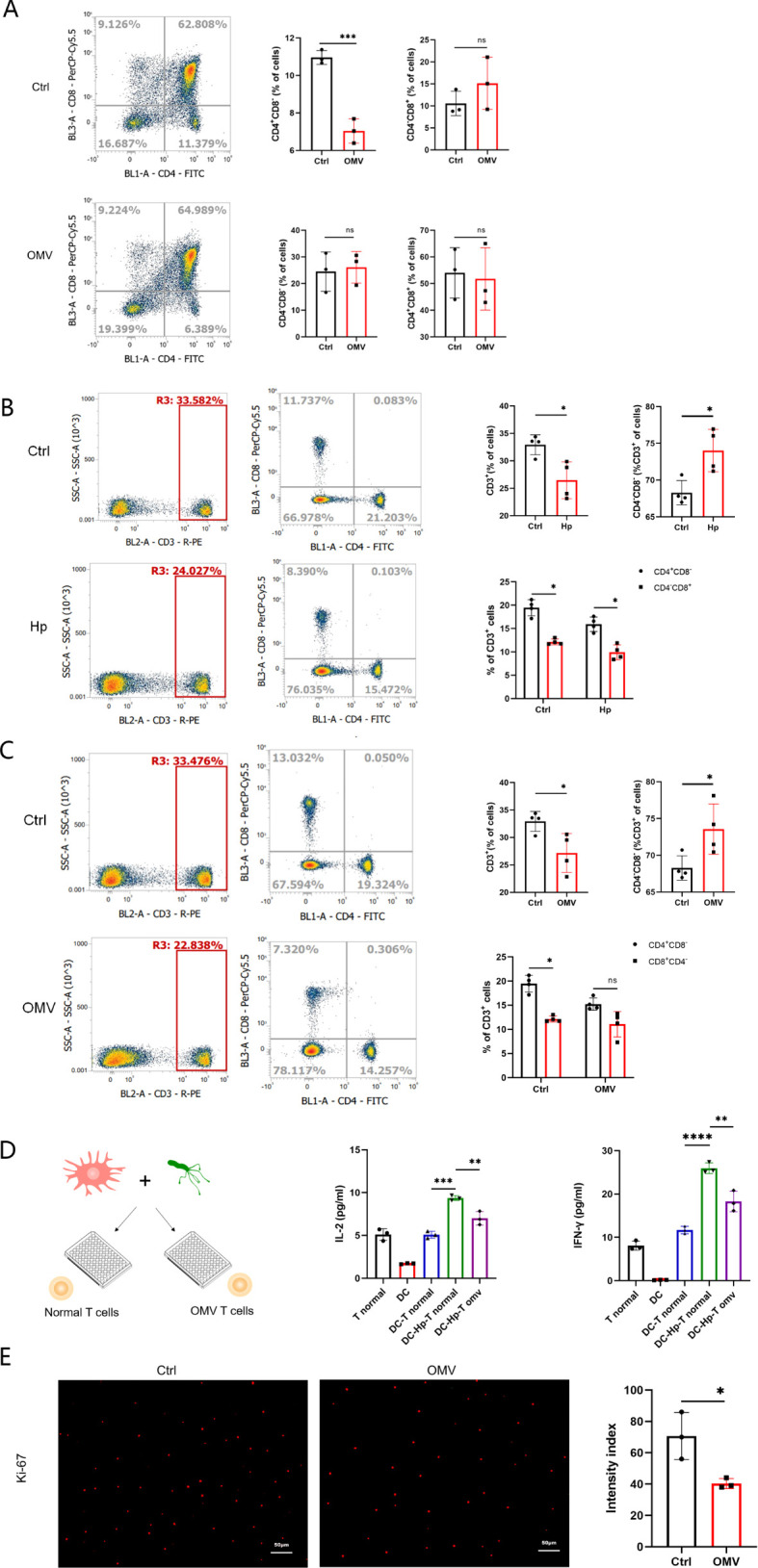
Induction by *H.pylori* OMVs of changes in T cell subsets in fetal mice thymus and peripheral blood in offspring mice. **(A)**, T cell subsets (CD4^+^CD8^-^, CD4^-^CD8^+^, CD4^+^CD8^+^, CD4^-^CD8^-^) in the thymus of fetal mice were detected by flow cytometry; results are expressed as mean ± SD and statistically tested by unpaired two-tailed t tests. **(B)**, T cell subsets (CD3^+^, CD3^+^CD4^+^CD8^-^, CD3^+^CD4^-^CD8^+^, CD3^+^CD4^-^CD8^-^) in peripheral blood of offspring mice born to mice experiencing *H.pylori* infection during pregnancy were detected by flow cytometry, 4 mice/group (n=4); results are expressed as mean ± SD, and statistical analysis was performed by unpaired two-tailed t test. **(C)**, Flow cytometry was used to detect the peripheral blood T cell subsets (CD3^+^, CD3^+^CD4^+^CD8^-^, CD3^+^CD4^-^CD8^+^, CD3^+^CD4^-^CD8^-^) in the offspring of E18-E19 pregnant mice injected with *H.pylori* OMVs via the tail vein, 4 mice/group (n=4); results are expressed as mean ± SD, and statistical analysis was performed by unpaired two-tailed t test. **(D)**, Expression of IL-2 and IFN-γ in the supernatants of mixed lymphocyte reaction cells was measured by ELISA, repeated three times; results are expressed as mean ± SD and statistically tested by unpaired two-tailed t test. **(E)**, Cells stained with Ki-67 were observed by fluorescence microscopy (×10 eyepiece, ×20 objective), scale: images were randomly collected three times and analyzed in a blind manner; results were expressed as mean ± SD and statistically tested by unpaired two-tailed t test. *P< 0.05, **P < 0.01, ***P < 0.001, ****P < 0.0001, ns, no significance..

We first examined the effect of *H.pylori* OMVs stimulation on T cell subsets in fetal mice thymus cultured *in vitro*. Compared with the control group, CD4^+^CD8^-^ T cells in the *H.pylori* OMVs stimulation group decreased significantly, whereas CD4^-^CD8^+^, CD4^+^CD8^+^, and CD4^-^CD8^-^ T cells did not change significantly (**Figure 5A**).

Next, we investigated the changes of T cells in the offspring of pregnant mice infected with *H.pylori*. The results indicated abnormal variations in the T cell subpopulations of peripheral blood in the offspring mice. Specifically, there was a significant reduction in CD3^+^, CD3^+^CD4^+^CD8^-^, and CD3^+^CD4^-^CD8^+^ T cells, while CD3^+^CD4^-^CD8^-^ T cells increased significantly (**Figure 5B**).

Then, we determined the changes of T cells in offspring of mice injected with *H.pylori* OMVs via the tail vein during pregnancy (E18-19). The results showed that the peripheral blood T cell subsets of offspring mice had abnormal changes, and the CD3^+^; and CD3^+^CD4^+^CD8^-^ T cells were significantly reduce, while CD3^+^CD4^-^CD8^-^ T cells increased significantly (**Figure 5C**). Subsequently, the ability of CD3^+^CD4^+^CD8^+^ T cells from the offspring mice to respond to *H.pylori* stimulation was verified. The production of IL-2 and IFN-γ by T cells from the offspring of the above model mice was shown to be significantly lower than that of the normal group (**Figure 5D**), and the proliferation ability of T cells was significantly lower than that of the control group (**Figure 5E**).

### 3.5 Molecular mechanism responsible for effects of *H.pylori* OMVs on thymic development in fetal mice

**Figure 6:**
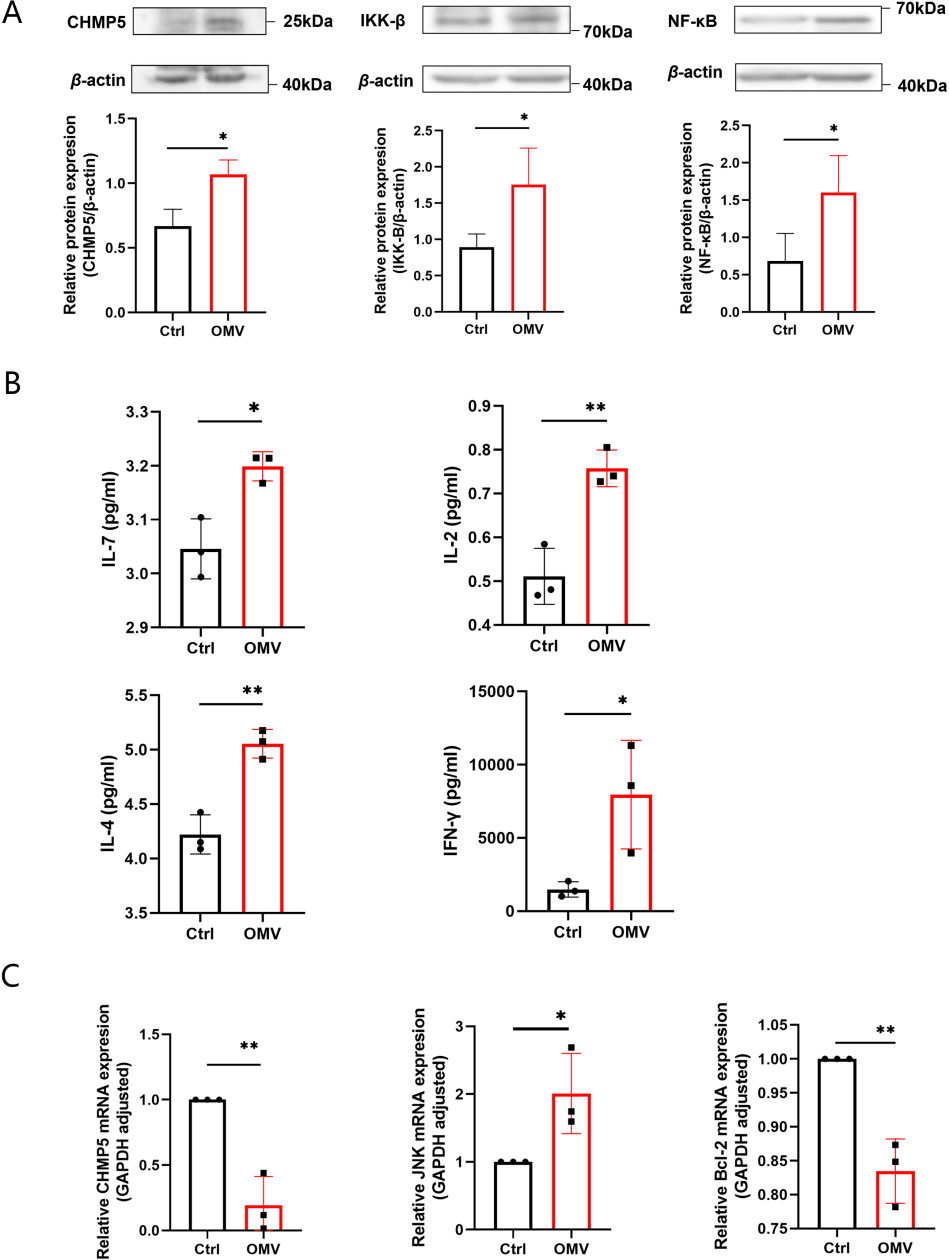
Induction by *H.pylori* OMVs of changes in signaling molecules related to T cell development in fetal mice thymus and their relationships. On day 0, the thymus of E15-16 fetal mice was stimulated with *H.pylori* OMVs: **(A)**, Western blot analysis showing the expression of CHMP5, IKK-β, and NF-κB; **(B)**, the expression levels of cytokines IL-7, IL-2, IL-4, and IFN-γ as determined by ELISA. **(C)**, After stimulation with *H.pylori* OMVs in the thymus of E15-16 fetal mice on day 3 of culture, qRT-PCR showing the expression of JNK, CHMP5 and Bcl-2 mRNA. Each independent experiment was repeated three times; results are presented as mean ± SD. Statistical tests were performed using an unpaired two-tailed t test. *P< 0.05, **P < 0.01.

**Figure 7:**
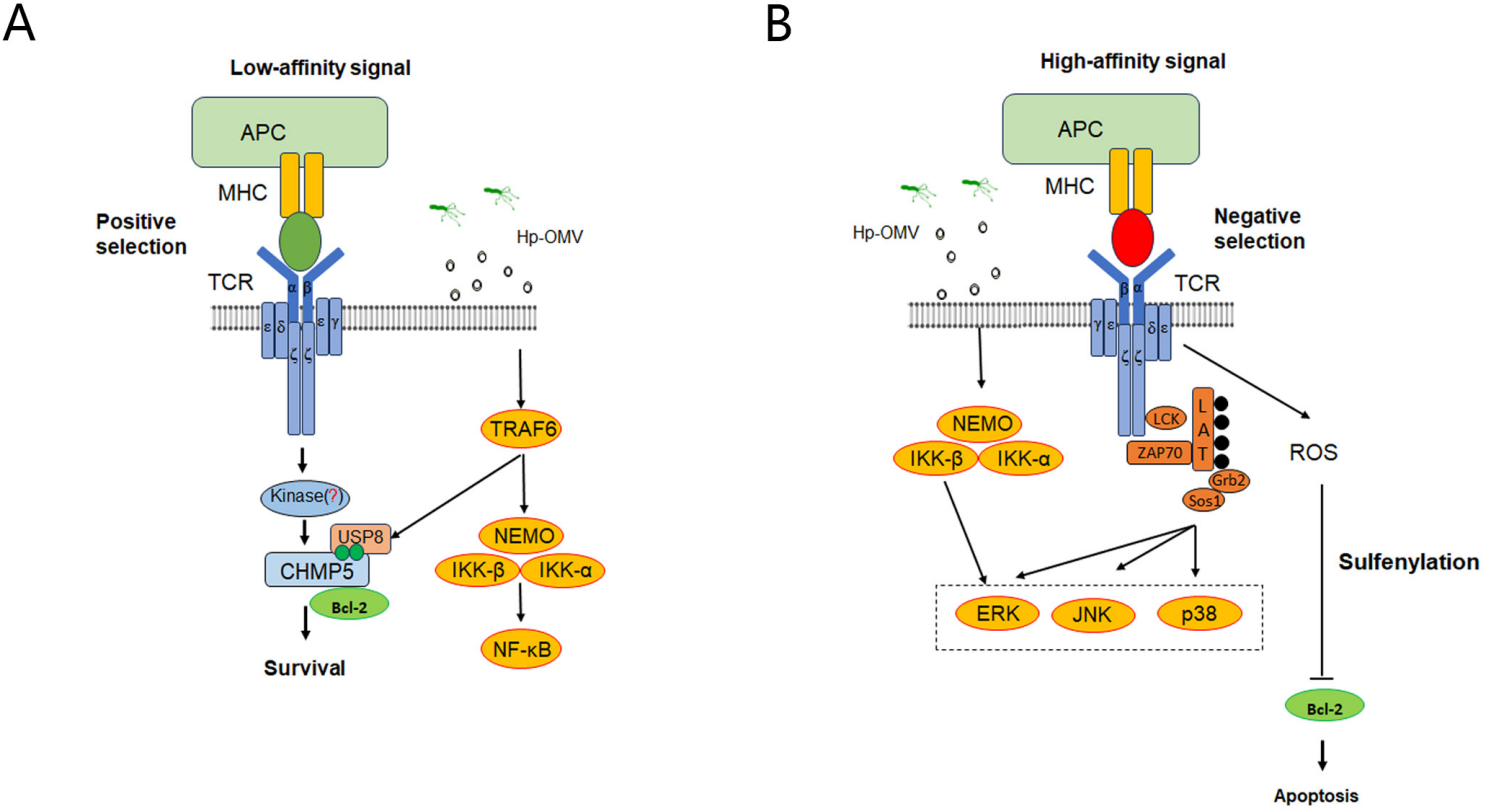
Possible mechanism of central tolerance of thymic T cells induced by *H.pylori* OMVs. **(A)**, During the positive selection phase, crosstalk might occur between the OMV activation pathway and the MHC pathway, which might regulate the stability of CHMP5 protein and promote the development of positively selected T cells. **(B)**, During the negative selection phase, crosstalk might occur between the OMV activation pathway and the TCR signaling pathway to promote T cell apoptosis. *P < 0.05, ** P < 0.01, *** P < 0.001, ns: no significance.

To explore the molecular mechanism responsible for the effects of *H.pylori* OMVs on thymus development, we tested positive selection and negative selection phase. In the positive selection phase, the expression levels of CHMP5, IKK-β, and NF-κB proteins, as detected by Western blotting, in the thymus tissues of E15-16 fetal mice were shown to be up-regulated (**Figure 6A**). ELISA revealed that the levels of IL-7, IL-2, IL-4 and IFN-γ in the supernatants were increased (**Figure 6B**). In the negative selection phase, the gene expression levels of CHMP5, JNK, and Bcl-2 in the thymus tissues of the 3-day stimulation group were examined by qRT-PCR: JNK expression was found to be significantly up-regulated, whereas CHMP5 and Bcl-2 expression was significantly down-regulated (**Figure 6C**). The above results thus showed that thymic stromal cells and related TCR and MHC signaling pathways were activated by *H.pylori* OMVs, and that the expression level of CHMP5 was changed in a bidirectional manner (**Figure 7**).

## 4 Discussion

In recent years, *H.pylori* OMVs have attracted much attention in the field of medical research (30–33). As an important class of microbial components, OMVs play a key role in bacteria-host interactions (34). In addition to its colonization and pathogenic role in the gastrointestinal tract, the *H.pylori* OMVs are able to cross a variety of biological barriers, including the gastric mucosa and the blood circulatory system (11, 33, 35). The background of this study builds on these findings and further explores the potential impact of *H pylori* OMVs during pregnancy, specifically whether they are able to cross placental barriers and affect the immune development of the offspring. This study provides the first direct evidence that *H pylori* OMVs are able to cross the placental barrier. In addition, the transfer of OMVs from mother to fetus is associated with T cell development.

It has been found that infection with *H pylori* during pregnancy leads to significant changes in T cell subsets in the offspring (36), specifically demonstrated by a decrease in the number of CD3^+^ T cells, which may affect the immune response and the establishment of tolerance in the offspring (37). Additionally, the increase in CD3^+^CD4^-^CD8^-^ T cells may be related to the immune response activated by maternal infection with *H pylori* or exposure to OMVs. These inflammatory signals could be transmitted to the fetus through the placenta, thereby affecting the development of its immune system. The elevation of the CD3^+^CD4^-^CD8^-^ T cells population might reflect an adaptive response of the fetal immune system to maternal infection, such as maintaining immune homeostasis through Th cell subsets. Although these CD3^+^ T cells, lacking both CD4^+^ and CD8^+^ coreceptors, account for only 3-5% of peripheral blood T lymphocytes, they possess both innate and adaptive immune functions (38). They play a crucial role in immune surveillance, infection resistance, and immune regulation. Abnormalities in their numbers may either weaken the effective defense against pathogens or increase the risk of autoimmunity due to long-term immune activation, potentially forming a synergistic effect with the Th2 bias immune characteristics of the neonatal period (39, 40). *H pylori* infection during pregnancy may affect T cell maturation and function through antigens transmitted by OMVs, thereby influencing the long-term immune health of the offspring. The reduction in the number and function of CD4^+^ T cells could have profound effects on the offspring’s immune function, including decreased defense against pathogens, diminished vaccination efficacy, and increased susceptibility to autoimmune and allergic diseases (41, 42).Understanding these nuances is essential for optimizing vaccine strategies and managing infectious diseases in neonates and infants (43).

Given the observed alterations in the T cell subsets of offspring, we hypothesize that *H.pylori* OMVs may act as a vector for the transmission of maternal antigens to the offspring, thereby impacting the development and function of offspring T cells. *H.pylori* OMVs possess the capability to traverse the placental barrier and deliver *H.pylori* antigens to the fetus. These antigens may then be processed and presented within the fetal thymus, influencing both positive and negative T cell selection processes. This sequence of events may ultimately lead to the establishment of central tolerance to *H.pylori* within the offspring T cells, subsequently modulating their immune response to *H.pylori*. This hypothesis is consistent with the well - characterized properties of *H.pylori* OMVs. For instance, *H.pylori* OMVs are known to carry a diverse array of bacterial proteins and toxins, and they play a crucial role in mediating bacterial-host interactions. Their unique ability to encapsulate and transport various biomolecules, along with their involvement in intercellular communication between bacteria and host cells, provides a mechanistic basis for the proposed role of *H.pylori* OMVs in the transfer of maternal antigens and the subsequent impact on offspring T cell development.

Our experimental results reveal that *H pylori* OMVs can penetrate the placental barrier. By injecting OMVs via the tail vein into pregnant mice at 18-19 days of gestation, it was found that OMVs were able to enter the offspring, confirming the possibility of OMVs crossing the placental barrier. In addition, the study observed changes in T-cell subsets in the offspring, suggesting that OMVs may play a role in the fetus, affecting its immune cell development and function. The placental penetration ability of OMVs may have an important impact on the immune development of the offspring. Placental barrier penetration may result in the exposure of the offspring to maternal *H pylori* antigens during the embryonic period, thereby affecting their T-cell development. This early antigenic exposure may lead to the development of central tolerance to *H pylori* in the offspring’s T cells, affecting their immune response to *H pylori* (44).

The study investigates the impact of *H pylori* OMVs on T cell development in the thymus. Continuous exposure to OMVs can lead to cross-presentation of *H pylori* antigen peptides by MHC class I molecules on thymic dendritic cells, affecting CD8^+^ T cell negative selection and potentially causing non-specific thymic damage if not cleared by antigen-presenting cells (36). Using embryonic thymus organ cultures, we found that OMVs upregulate IKK-β, CHMP5, and NF-κB during positive selection, promoting T cell survival through pathways involving NF-κB and CHMP5-stabilized Bcl-2. OMVs also increase cytokine expression (e.g., IL-2, IL-4, IL-7, IFN-γ), supporting T cell maturation. In negative selection, OMVs upregulate JNK while downregulating Bcl-2 and CHMP5, indicating crosstalk between OMV-activated pathways (e.g., TLRs) and TCR signaling. Further studies are needed to clarify the mechanisms, particularly the kinase responsible for CHMP5 phosphorylation (45).

The central tolerance induced by OMVs has profound implications for the design of *H pylori* vaccines. Central tolerance is a critical mechanism by which the immune system eliminates self-reactive T cells during development to prevent autoimmune diseases. However, antigens delivered by *H pylori* OMVs can induce central tolerance, thereby reducing vaccine efficacy. For instance, OMVs can activate TLR pathways and induce the production of immunosuppressive cytokines such as IL-10, limiting inflammatory responses and promoting bacterial survival (46). These immune-modulating mechanisms may dampen the immunogenicity of vaccines, especially if central tolerance is established during fetal development, rendering the immune system less responsive to *H pylori* antigens later in life.

To address this challenge, several vaccine design strategies should be considered. First, it is essential to choose antigens that are less likely to induce central tolerance. For example, epitope-based vaccines designed through immunoinformatics have demonstrated robust immunogenicity, capable of inducing strong cellular and humoral immune responses (47). Second, leveraging the natural immunogenic properties of OMVs, such as engineering OMVs to carry specific antigenic epitopes, can enhance antigen presentation efficiency and bypass central tolerance mechanisms (48). Additionally, combining novel adjuvants (e.g., OMVs with removed endotoxins) (49) or delivery systems (e.g., lipid nanoparticles) (50) can further enhance vaccine immunogenicity and safety.

Future research should focus on elucidating the mechanisms by which OMVs induce central tolerance and exploring strategies to overcome this challenge in vaccine design. Specifically, further investigation is needed to understand how OMVs modulate T cell selection and central tolerance via TLR pathways and TCR signaling. Moreover, developing novel vaccine platforms based on OMVs, such as glycoengineered OMVs or OMVs incorporating antigens from multiple pathogens, may provide innovative solutions for vaccine design. After validating the efficacy and safety of these vaccines in animal models, advancing them to clinical trials will be essential for developing effective vaccines against *H pylori* and other pathogens. In summary, by unraveling the immune-modulating mechanisms of OMVs and integrating modern vaccine technologies, it is possible to develop more effective vaccines that overcome the limitations imposed by central tolerance.

## 5 Conclusions

Our study indicates that *H pylori* OMVs can cross the placental barrier, inducing central tolerance in offspring T cells to the bacteria. This finding reveals a significant pathogenic mechanism and underscores the need for vaccine strategies to consider central tolerance antigens. However, the specific mechanisms of OMVs influence on T cell tolerance require further investigation, acknowledging the limitations in fully elucidating the impact of OMVs on immune development. Future research should address these gaps to enhance our understanding and develop effective *H pylori* vaccines.

## Data Availability

The original contributions presented in the study are included in the article/**Supplementary Material**. Further inquiries can be directed to the corresponding authors.
