# Supplementary material for: Helicobacter pylori outer membrane vesicles mediate central tolerance in C57BL/6J mice offspring T cells via maternal-fetal transmission
Source: Front Immunol. 2025 Apr 15;16:1522842. doi: 10.3389/fimmu.2025.1522842 (PMC12037491; doi:10.3389/fimmu.2025.1522842)
Supplement: Supplementary file 1 [file DataSheet1.pdf]

Supplementary Figure

Peripheral blood flow results of offspring mice

Ctrl 1

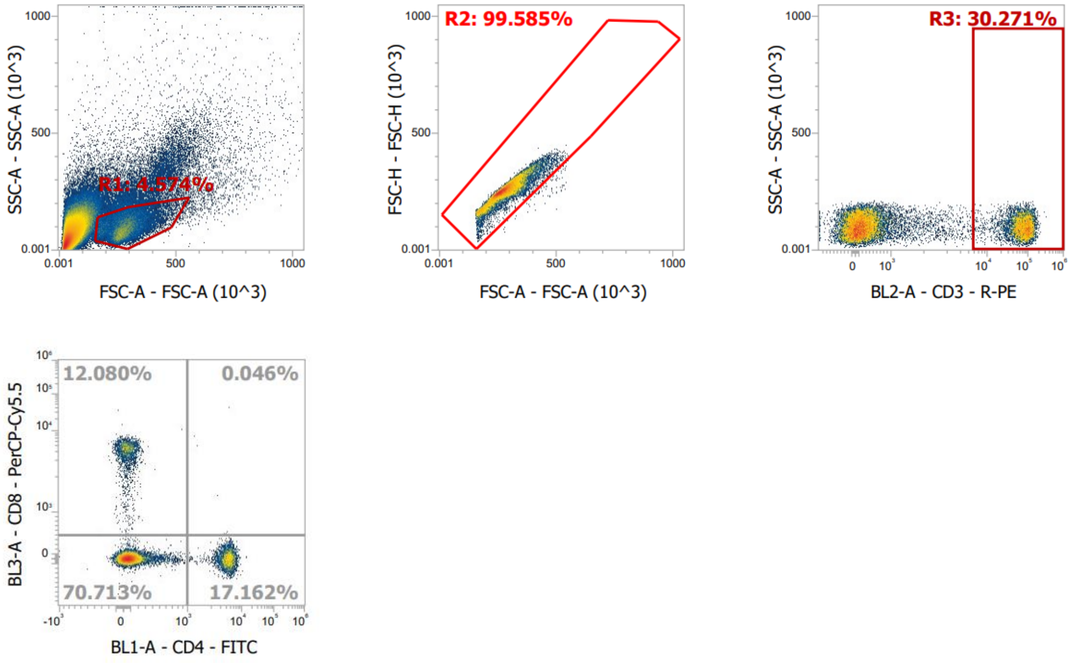

Ctrl 2

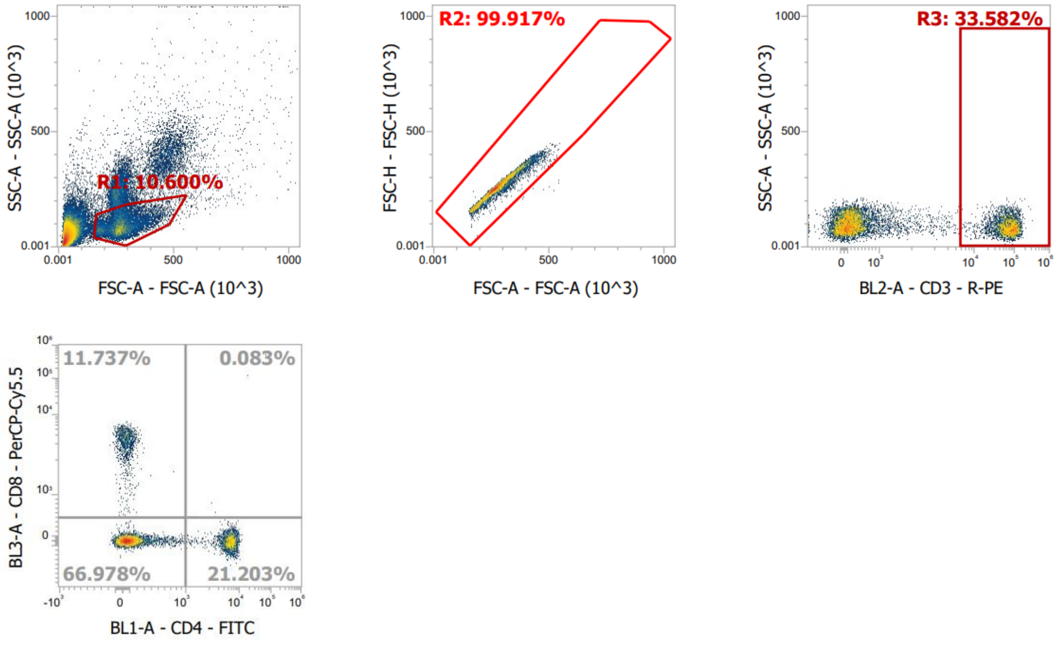

Ctrl 3

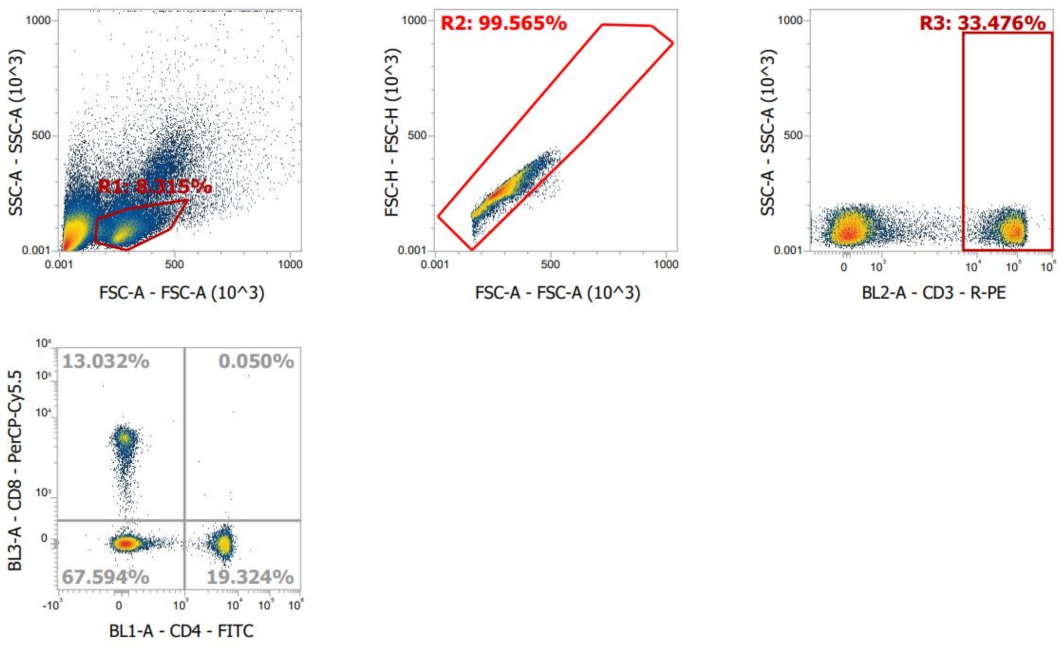
R1: 8.315%
SSC-A - SSC-A (10^3)
FSC-A - FSC-A (10^3)
R2: 99.565%
FSC-H - FSC-H (10^3)
FSC-A - FSC-A (10^3)
R3: 33.476%
SSC-A - SSC-A (10^3)
BL2-A - CD3 - R-PE
13.032%
0.050%
67.594%
19.324%
BL3-A - CD8 - PerCP-Cy5.5
BL1-A - CD4 - FITC

Ctrl 4

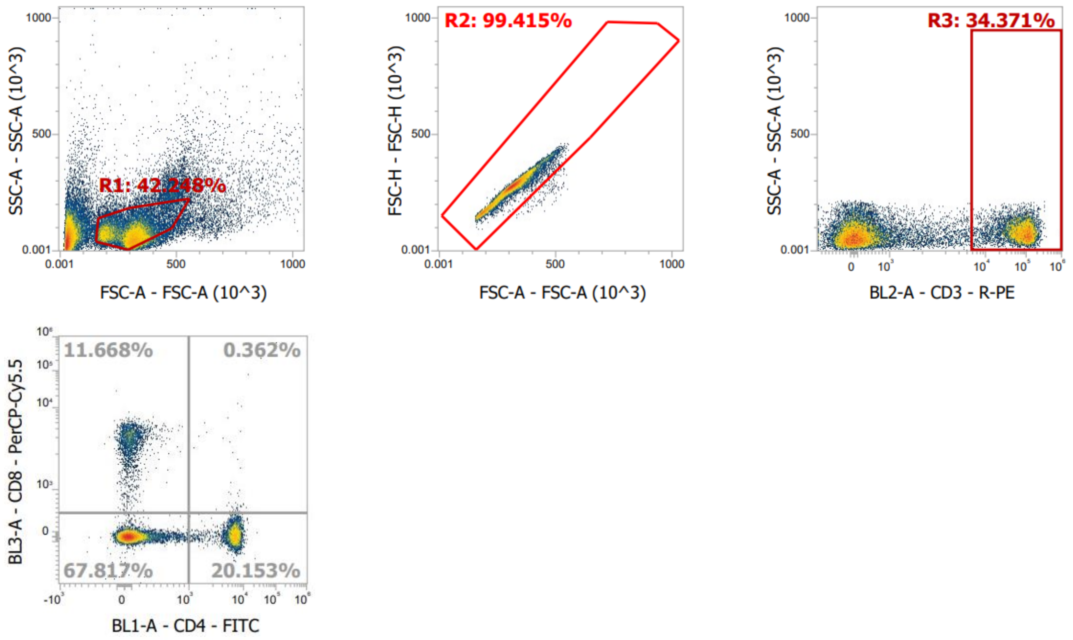
R1: 42.248%
SSC-A - SSC-A (10^3)
FSC-A - FSC-A (10^3)
R2: 99.415%
FSC-H - FSC-H (10^3)
FSC-A - FSC-A (10^3)
R3: 34.371%
SSC-A - SSC-A (10^3)
BL2-A - CD3 - R-PE
11.668%
0.362%
67.817%
20.153%
BL3-A - CD8 - PerCP-Cy5.5
BL1-A - CD4 - FITC

Hp 1

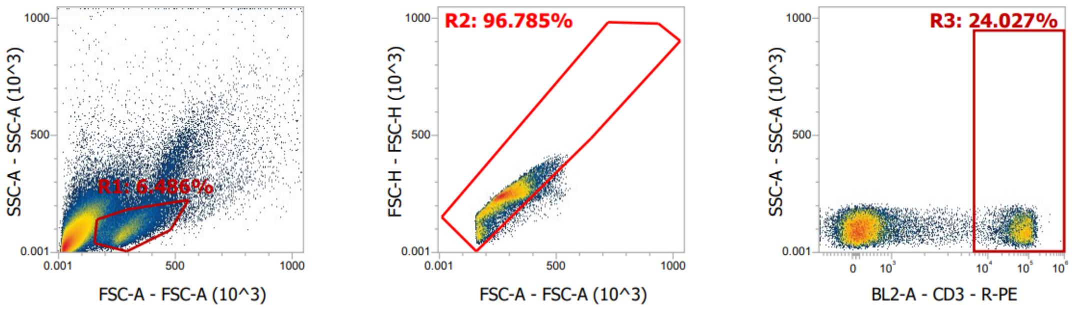
R1: 6.486%
SSC-A - SSC-A (10^3)
FSC-A - FSC-A (10^3)
R2: 96.785%
FSC-H - FSC-H (10^3)
FSC-A - FSC-A (10^3)
R3: 24.027%
SSC-A - SSC-A (10^3)
BL2-A - CD3 - R-PE

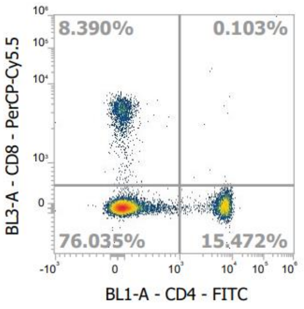
8.390%
0.103%
76.035%
15.472%
BL3-A - CD8 - PerCP-Cy5.5
BL1-A - CD4 - FITC

Hp 2

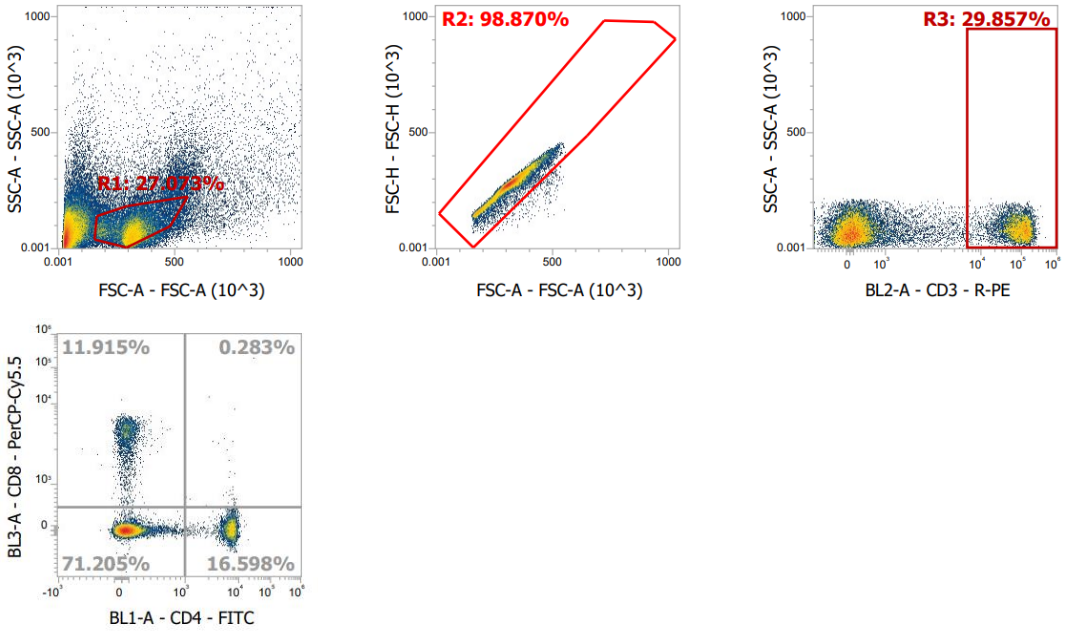
R1: 27.073%
SSC-A - SSC-A (10^3)
FSC-A - FSC-A (10^3)
R2: 98.870%
FSC-H - FSC-H (10^3)
FSC-A - FSC-A (10^3)
R3: 29.857%
SSC-A - SSC-A (10^3)
BL2-A - CD3 - R-PE
11.915%
0.283%
71.205%
16.598%
BL3-A - CD8 - PerCP-Cy5.5
BL1-A - CD4 - FITC

Hp 3

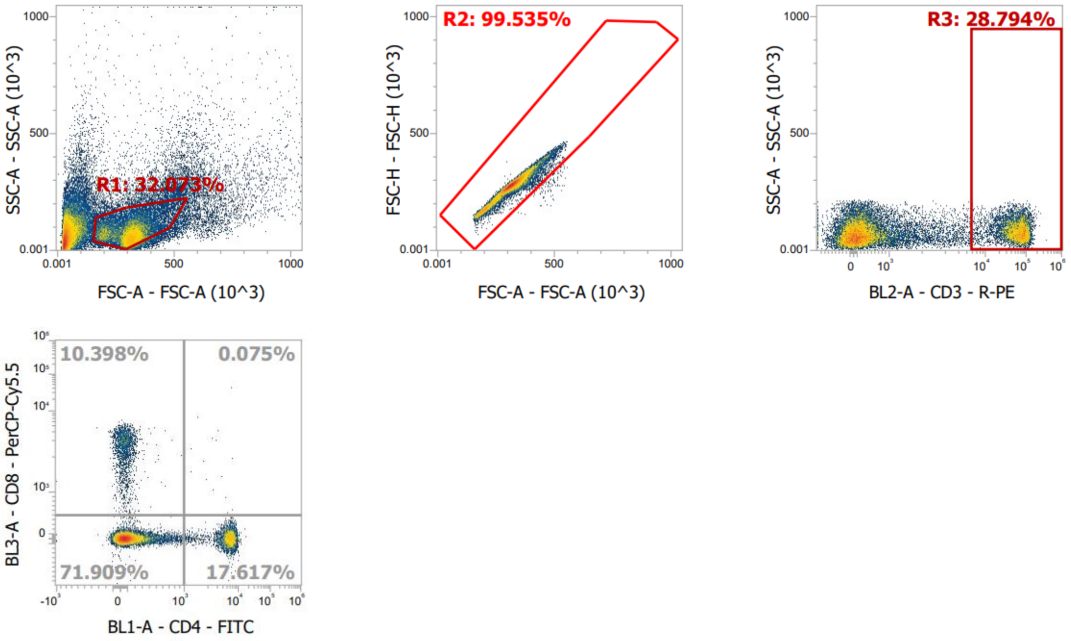
R1: 32.073%
SSC-A - SSC-A (10^3)
FSC-A - FSC-A (10^3)
R2: 99.535%
FSC-H - FSC-H (10^3)
FSC-A - FSC-A (10^3)
R3: 28.794%
SSC-A - SSC-A (10^3)
BL2-A - CD3 - R-PE
10.398%
0.075%
71.909%
17.617%
BL3-A - CD8 - PerCP-Cy5.5
BL1-A - CD4 - FITC

Hp 4

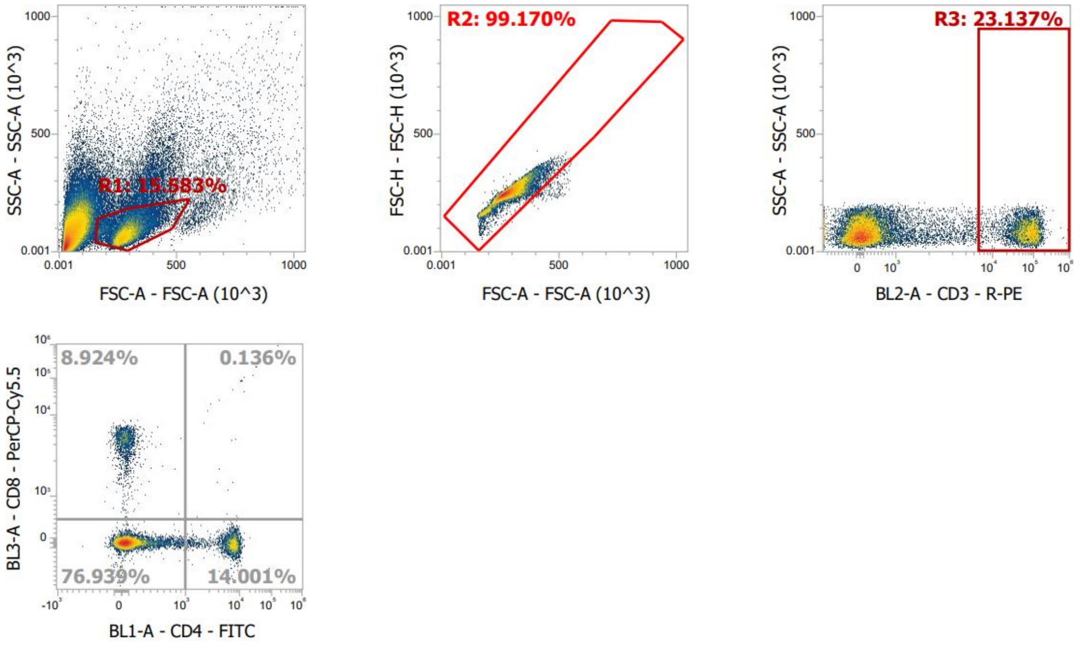

OMV 1

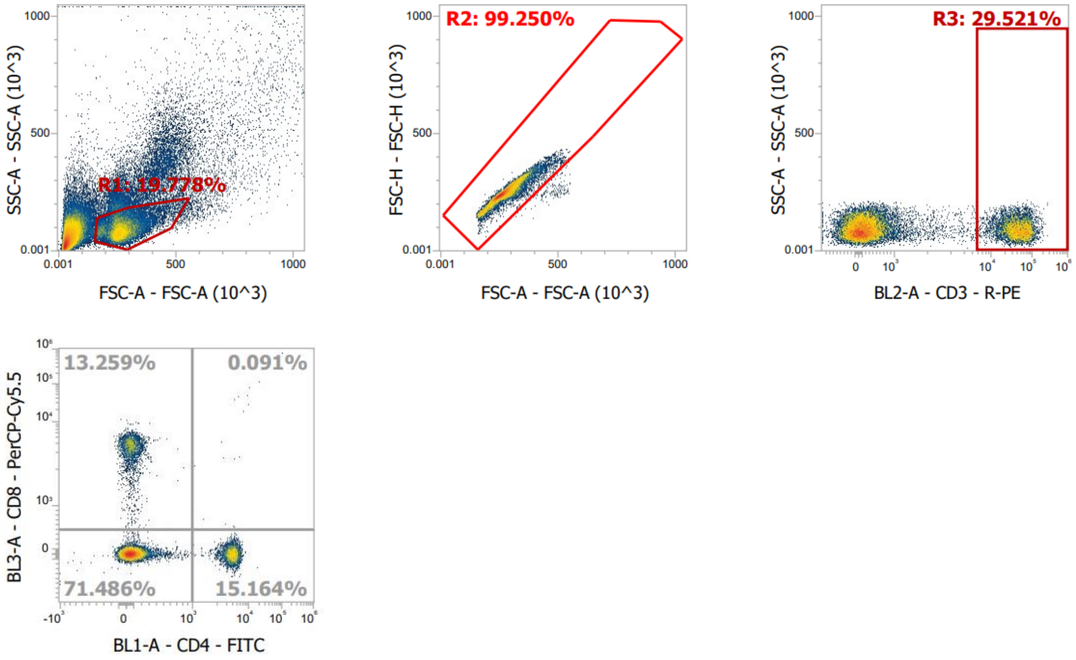

OMV 2

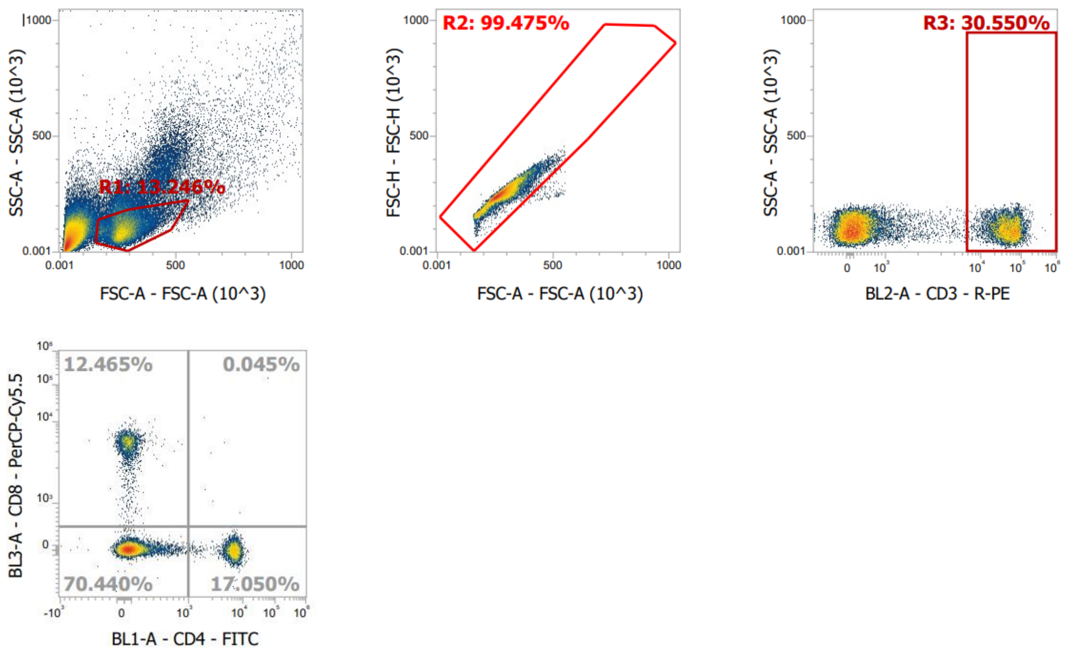
SSC-A - SSC-A (10^3)
FSC-A - FSC-A (10^3)
R1: 13.246%
FSC-H - FSC-H (10^3)
R2: 99.475%
BL2-A - CD3 - R-PE
R3: 30.550%
BL3-A - CD8 - PerCP-Cy5.5
BL1-A - CD4 - FITC
12.465%
0.045%
70.440%
17.050%

OMV 3

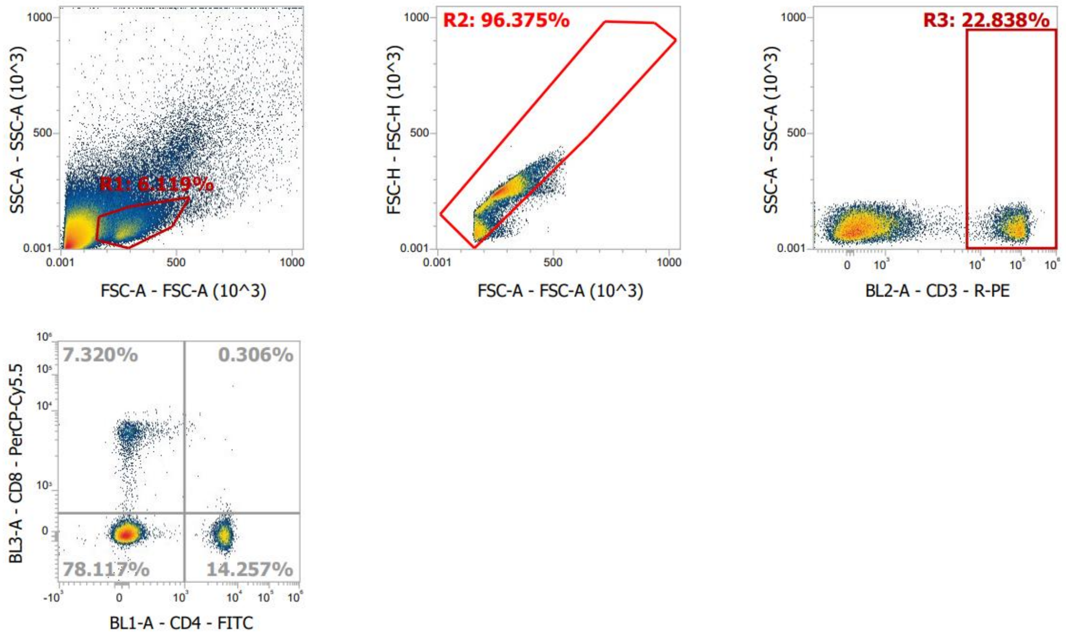
SSC-A - SSC-A (10^3)
FSC-A - FSC-A (10^3)
R1: 6.119%
FSC-H - FSC-H (10^3)
R2: 96.375%
BL2-A - CD3 - R-PE
R3: 22.838%
BL3-A - CD8 - PerCP-Cy5.5
BL1-A - CD4 - FITC
7.320%
0.306%
78.117%
14.257%

OMV 4

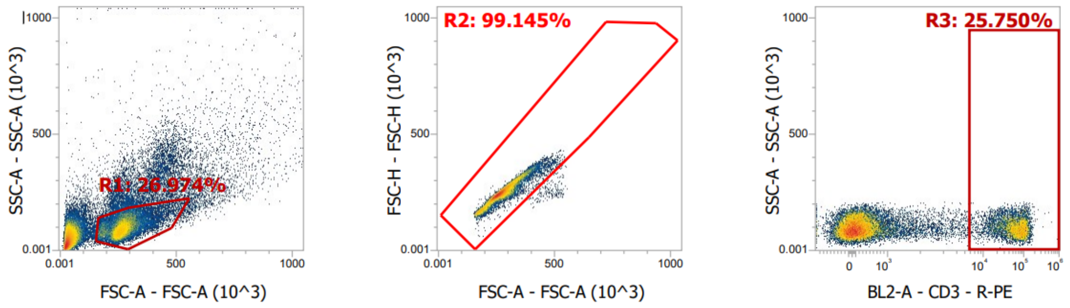
SSC-A - SSC-A (10^3)
FSC-A - FSC-A (10^3)
R1: 26.974%
FSC-H - FSC-H (10^3)
R2: 99.145%
BL2-A - CD3 - R-PE
R3: 25.750%

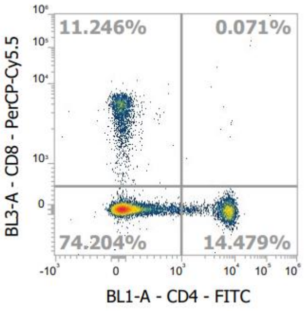
11.246%
0.071%
74.204%
14.479%
BL3-A - CD8 - PerCP-Cy5.5
BL1-A - CD4 - FITC
